# Indoor Air Quality Analysis of Newly Built Houses

**DOI:** 10.3390/ijerph16214142

**Published:** 2019-10-28

**Authors:** Norimichi Suzuki, Hiroko Nakaoka, Masamichi Hanazato, Yoshitake Nakayama, Kayo Tsumura, Kazunari Takaya, Emiko Todaka, Chisato Mori

**Affiliations:** 1 Center for Preventive Medical Sciences, Chiba University, 6-2-1 Kashiwanoha, Kashiwa, Chiba 277-0882, Japan; hnakaoka@faculty.chiba-u.jp (H.N.); hanazato@chiba-u.jp (M.H.); seiken@chiba-u.jp (Y.N.); tsumu-kayo@chiba-u.jp (K.T.); todakae@faculty.chiba-u.jp (E.T.); cmori@faculty.chiba-u.jp (C.M.); 2 National Institute of Occupational Safety and Health, 6-21-1 Nagao, Tama-ku, Kawasaki, 214-8585, Japan; takaya-k@h.jniosh.johas.go.jp; 3 Center for Preventive Medical Sciences, Chiba University, 1-8-1 Inohana, Chuo-ku, Chiba-shi, Graduate School of Medicine, Chiba University, Chiba 260-8672, Japan

**Keywords:** indoor air quality, volatile organic compounds, total volatile organic compounds, newly built houses, sick building syndrome

## Abstract

Recently, people have become increasingly aware of potential health issues related to indoor environments. In this study, we measure the concentrations of various volatile organic compounds, carbonyl compounds, and semi-volatile organic compounds, as well as the ventilation rates, in 49 new houses with light-gauge steel structures one week after completion. The proper indoor air quality of new residential environments can be ensured by characterizing people’s exposure to certain chemicals and assessing future risks. Our results show that the concentrations of the measured compounds were lower than the guideline values set by the Ministry of Health, Labour and Welfare of Japan, and would continue to decrease. However, we observed that unregulated compounds, assumed to be substitutes for regulated solvents, contributed substantially to the total volatile organic compounds. To reduce indoor chemical exposure risks, the concentrations of these unregulated compounds should also be minimized. In addition, their sources need to be identified, and manufacture and use must be monitored. We believe it is important to select low-emission building materials for reducing residents’ exposure to indoor chemicals.

## 1. Introduction

Indoor air quality (IAQ) can have a significant impact on human health. Recently, there has been increasing concern regarding the adverse health effects of various chemicals released by construction materials and household items into indoor environments [1,2,3,4]. According to the previous studies, indoor air pollutants and microbiological contaminants pose public health risks [5,6,7,8]. Therefore, for the public’s safety, we should attempt to minimize their concentrations. They are suspected to be caused by volatile organic compounds (VOCs) emitted by building materials and other sources in newly built or refurbished houses.

Recent technological progress toward reducing household energy consumption has led to increased demand for highly airtight and insulated homes. However, making homes more airtight and heavily insulated have increased the likelihood of chemical compounds being retained indoors, resulting in increased air contamination [9,10]. Various chemical compounds are emitted into rooms by a wide range of sources, including building and internal materials, furniture, curtains, air fresheners, deodorants, pesticides, personal care products, cooking, and other everyday household products.

In particular, building materials and adhesives are important sources of VOC emissions, particularly in newly built houses [11,12]. It has been reported that current new-build houses have high concentrations of various VOCs emitted by building materials and products [13,14,15]. There has thus been increasing interest in changing new house designs and selecting suitable building materials. The European Union has published a list of substances and their associated emission limits, namely the European Union–Lowest Concentration of Interest (EU–LCI) values [16]. This gives the reference VOC concentrations.

European countries harmonize these values, but Japan currently does not provide labels or concentration limits based on the emissions from building materials. The only exception is materials containing formaldehyde, which are regulated by the revised Building Standard Law developed by the Japanese Ministry of Land, Infrastructure, Transport and Tourism (MLIT). In addition, in Japan, newly built houses are often delivered to clients immediately after completion. There is thus a risk that consumers will be exposed to high chemical concentrations if the indoor air of their newly built houses is contaminated, since new-build houses have high concentrations of various VOCs emitted by building materials and products [11,12]. In our previous study [17], we assessed IAQ by measuring the levels of five compounds (styrene, formaldehyde, ethylbenzene, toluene, and xylene) for which the MLIT’s Housing Quality Assurance Act sets out guideline values, as well as the total amount of VOCs (TVOC). Our results showed that it is possible to achieve good IAQ with low VOC concentrations, much lower than the Act’s guideline values, even just after construction is completed by using low-emission materials and maintaining ventilation rates of more than 0.5 air changes per hour, as required by Japanese law.

In this study, we collaborated with a house builder to focus on building houses with better IAQ. They attempt to build houses using materials that have low chemical compound emissions (based on emission rates calculated in small chamber method) and sell them as air-quality-conscious houses. To evaluate whether selecting low-emission materials and building products is an effective way to improve IAQ, we collected and analyzed air samples after the construction of these air-quality-conscious houses was complete but prior to occupancy.

We measured the concentrations of 55 VOCs, 14 carbonyl compounds (aldehydes and ketones), and 22 semi-volatile organic compounds (SVOCs) in 49 newly built houses soon after construction was complete (i.e., within a week). Then, we compared these results with the 13 guideline values and the interim TVOC target value set by the Ministry of Health, Labour and Welfare of Japan (MHLW) to assess the associated indoor air contamination risks. We also compared this data with the results of other studies and measured several environmental parameters, namely the ventilation rate, temperature, and relative humidity. The aim of this study is to investigate whether selecting and using low-emission construction materials can help to create low levels of VOCs with fewer chemicals, even just after completion.

## 2. Materials and Methods

### 2.1. House Characteristics

The houses considered in this study were built in Japan’s Chiba Prefecture by a construction company. We selected 89 houses from among those built between May 2014 and November 2015. Shortly after the houses were completed (immediately after the last finish nailing), we obtained permission from the houses’ owners to measure the levels of VOCs, carbonyl compounds, and SVOCs in the indoor air. A total of 49 owners gave their consent to conduct this study, and we used these houses as test sites.

The houses were constructed using light-gage steel with an average of 2.4 floors, and the mean living room and bedroom areas were 30.86 and 13.48 m^2^, respectively. The rooms were floored in laminate wood and the walls were either wallpapered using starch adhesives or painted using water-based paint. All the initial measurements were made while the rooms were devoid of furniture.

### 2.2. Indoor Air Sampling and Analysis

All measurements were performed according to “The standard methods of air sampling and measurement,” issued by the MHLW of Japan [18]. Before sampling the air, all the windows and doors were left open for more than 30 min for ventilation and were subsequently closed for more than 5 h. Then, air samples were collected from two rooms in the houses (living rooms and bedrooms) via active sampling for 60 min while their ventilation systems were operating, but with their air conditioning systems switched off. Simultaneously, several environmental factors were measured, namely the temperature, humidity, and ventilation rate. In Japan, the Building Standard Law stipulates mechanical ventilation equipment for all buildings and ventilation rate of 0.5 air changes per hour or more in residential rooms. The proposed study considers it as sufficient to measure one location per house for confirmation. Outdoor air samples were also collected every time at a point near each house under the eaves. Based on these samples, the levels of 55 VOCs, 14 carbonyl compounds, and 22 SVOCs were identified and quantified (Tables S1–S3 in the Supplementary Materials).

### 2.3. VOCs

Adsorbent tubes made of Tenax-TA^®^ (a porous polymer resin based on 2.6-diphenylene oxide) were obtained from Sigma-Aldrich (St. Louis, Missouri, USA) and used to actively sample the VOCs. Air samples were collected by pumping air through the tubes at a flow rate of 100 mL/min for 30 min, then analyzed using an Agilent 7890 B gas chromatograph (Agilent Technologies Inc., Tokyo, Japan) equipped with an MSD 5977A quadrupole mass spectrometer and a TurboMatrix ATD 650 TD unit (PerkinElmer Japan Co. Ltd., Tokyo, Japan). Next, thermal desorption measurements were carried out by heating the Tenax-TA for 10 min at 280 °C. Here, the split ratio was 20:1 and the transfer-line temperature was 230 °C.

Next, DB-1 columns (30 m × 0.25 mm; i.d.: 1.0 µm; Agilent Technologies Inc.) were used to separate the samples at 35 °C for 5 min, before the columns were heated to 240 °C at a rate of 10 °C/min. Helium (more than 99.9999% pure) was used as the gas chromatography (GC) carrier gas, flowing into the columns at a rate of 1.0 mL min^−1^. The VOCs were identified and quantified in scan mode at mass-to-charge (*m*/*z*) ratios in the 40–350 range. The concentrations of 55 compounds were calculated based on their individual response factors. Calibration was accomplished using two different concentrations, i.e., 5–50 ppm, and linear responses were obtained. Finally, the TVOC was calculated by summing the amounts of all the VOCs, carbonyl compounds, and SVOCs.

### 2.4. Carbonyl Compounds

First, 2,4-dinitrophenylhydrazine (DNPH) active gas tubes (designed for aldehydes and ketones; Shibata Scientific Technology Ltd., Saitama, Japan) were used to calibrate the carbonyl compound measurements. The samples were collected over 30 min with a pump flow rate of 1.0 L/min, and then the gas tubes were eluted at a flow rate of 2–5 mL/min using a 10-mL syringe filled with acetonitrile. High-performance liquid chromatography analyses were conducted with 5.0 mL samples, using a prominence UFLC from Shimadzu Corporation (Kyoto, Japan) equipped with 2 LC-20AD liquid feed pumps, a SIL-20AC auto-sampler, and an SPD M20A photodiode array detector. Ascentis RP-Amide columns (150 mm × 4.6 mm; i.d.: 2.7 µm; Sigma-Aldrich) were used for separation, the column oven temperature was 40 °C, and the injection volume was 10 µL. The mobile phases were acetonitrile/water solutions with ratios of 40:60 (Solution A) and 80:20 (Solution B). The column flow rate was 1.0 mL/min, using 100% of Solution A for the first 30 min, followed by 75% of Solution A and 25% of Solution B for 55 min, and finally 100% of Solution B for 5 min. The concentrations of all the compounds were calculated using the corresponding standards. Calibration was accomplished with a concentration of 1 ppm.

### 2.5. SVOCs

To calibrate the SVOC measurements, 47-mm Empore C-18 FF Disks (3M Japan Ltd. Two Harbors, Minnesota, USA) in an EMO holder (GL Sciences, Inc., Tokyo, Japan) were preconditioned with acetone. The samples were collected over 120 min with a pump flow rate of 10 L/min, then the filters were transferred to 10-mL stoppered test tubes, to which 7 mL of acetone and 1 μL of a 100-μg/mL internal standard solution (acenaphthene-d10) were added. After 5 min of ultrasonic extraction, the samples were centrifuged for a further 5 min at 2500 rpm. Then, the supernatants were transferred to different test tubes and concentrated to 1.0 mL under a nitrogen stream (60 °C), before their contents were analyzed via GC/mass spectrometry using an MSD 5977A quadrupole mass spectrometer equipped with an Agilent 7890 B gas chromatograph and a 7693 auto-sampler (Agilent Technologies Inc., Santa Clara, California, USA). A DB-1 column (30 m × 0.25 mm; i.d.: 1.0 µm; Agilent Technologies Inc.) was used for separation, and its temperature was increased from 60 °C to 280 °C at a rate of 12 °C/min, maintaining the final temperature for 10 min. The injector temperature was 280 °C and 1.0 µL volumes were injected in splitless mode. Helium (more than 99.9999% pure) was used as the carrier gas, with a flow rate of 1.0 mL/min. The analysis was performed in SIM mode. The concentrations of all the compounds were calculated using the corresponding standards. Calibration was accomplished using two different concentrations, 1–100 ppb and linear responses were obtained.

### 2.6. Ventilation Rate

The ventilation rates in the living rooms were calculated using the concentration attenuation method with a tracer gas (CO_2_). Dry ice was placed indoors and stirred using a turbo circulator. After confirming that the CO_2_ concentration was stable with a CO_2_ densitometer (TES-1370, Satoshoji Digital, Kanagawa, Japan), the maximum value was recorded and the concentration measured at one-minute intervals as it gradually decreased over a period of 60 min. Based on the initial (maximum) value, the ventilation rate *V* was calculated according to the formula given in the “Standard Methods of Analysis for Hygienic Chemists” ([19]):

*V* = 2.303 × *VR*/*t* × log {(C_1_ − C_0_) / (*C_t_* − C_0_)} (1)

where *VR* is the room volume (m^3^), *t* is the duration of the mechanical ventilation’s operation, *C_t_* is the tracer gas concentration (µg/m^3^ or mL/m^3^) at time *t*, *C*_1_ is the initial tracer gas concentration (µg/m³ or mL/m^3^), and *C*_0_ is the outdoor tracer gas concentration (µg/m³ or mL/m^3^).

## 3. Results

### 3.1. Indoor Environment Characteristics

Table 1 lists the mean, median, maximum, and minimum ventilation rates together with the temperatures and relative humidity levels for the indoor environments. The temperature varied from 7.3 °C to 31.3 °C in the living rooms and from 8.0 °C to 33.2 °C in bedrooms, whereas the relative humidity varied from 24.6% to 82.2% in the living rooms and 24.4% to 77.5% in bedrooms. Notably, the living room ventilation rates ranged from 0.5–2.3 per hour, meaning that all the tested rooms exceeded the standard (0.5 air changes per hour) specified in the amended Building Standard Law set by the MLIT of Japan [20].

### 3.2. Chemical Compound Concentrations

The MHLW of Japan has set non-binding guideline limits on the concentrations of 13 chemicals, as well as an interim TVOC target of 400 μg/m^3^. Table 2 shows limits of quantitation (LoQs), and guideline values for these 13 compounds. In this study, we measured the levels of these compounds and compared them with the guideline values. Table 2 also shows the mean, median, maximum, and minimum concentrations, together with the frequencies with which they were detected in the test houses and the TVOC. In all cases, the concentrations of these 13 compounds were below the recommended limits. Of the 13 compounds, the three most abundant were acetaldehyde, toluene, and formaldehyde, whose mean (±SD) living room concentrations were 24 ± 10, 12 ± 11 and 15 ± 6.5 μg/m^3^, respectively. These three compounds were also detected in all the bedrooms. The mean outdoor TVOC level was 85.0 µg/m^3^ and was always low regardless of temperature and humidity.

## 4. Discussion

In this study, we investigated the IAQ of newly built houses in terms of the concentrations of 55 VOCs, 14 carbonyl compounds, and 22 SVOCs, via active sampling. The VOC measurements of outdoor air were conducted at the same time as those in indoor air; however, the concentration levels of outdoor air were always very low. Indoor air is strongly affected by outside air, but the results led us to assume that indoor air pollutants had been emitted from indoor materials. Here, we focused purely on the effects of building materials on air quality, excluding furniture and other household items. We measured the concentrations of a variety of chemical compounds in 49 houses immediately after construction was complete (i.e., within a week). At this time, the concentrations of the 13 compounds for which the MHLW of Japan has set guidelines were generally at a tenth of the guideline levels or below. However, the TVOC of several samples exceeded the provisional target of 400 μg/m^3^. We believe this was because these measurements were conducted in summer and the maximum temperature was very high (31.3–33.2 °C). In addition, they were performed less than a week after completion, within one to three days. The relation between the temperature and concentration levels of TVOC was calculated with Spearman’s rank correlation coefficient. There was a slight positive correlation (r = 0.390 and *p* = 0.006 for the living rooms and r = 0.298 and *p* = 0.042 for the bedrooms). There were several samples in which the TVOC levels exceeded the provisional target value of 400 μg/m^3^. We have assumed that this excess value can be attributed to these measurements being conducted during the summer when the maximum temperature was very high (31.3–33.2 °C). However, almost all concentration levels of TVOC (Table 2) could be kept low with a mean of 389 μg/m^3^ and median of 390 μg/m^3^ in the living rooms and those in the bedrooms can be kept as 389 and 390 μg/m^3^, respectively, even when the room temperatures were high.

In addition, we compared the values for the 13 VOCs and the TVOC obtained in this study with the results of other studies, by Park and Ikeda [21], Järnström et al. [13], Shin and Jo [22], Noguchi et al. [23], and Onuki et al. [14], that were conducted on newly built, less than a year old, or unoccupied dwellings (Table 3). Note that the 13 compounds considered here include some SVOCs, which must be sampled and analyzed separately, and this SVOC data was not provided by the other studies. This comparison shows that almost all the VOC and TVOC values seen in this study are lower than those of the other studies. It was difficult to implement conditions, such as measurement procedures, apparatuses, and temperatures, which were similar to those of other studies. Therefore, our comparison may lack accuracy, which would be a limitation of this study. However, we did try to make our conditions, such as higher temperatures and shorter times before measurements, severer than those of the other studies. 

Onuki et al. investigated the IAQ of houses built in Japan between 2007 and 2009, within six months of their completion [14]. They found that the maximum concentrations of some compounds exceeded the guideline values, and were much higher than the values observed in our study. In addition, Noguchi et al. measured the IAQ of a daycare center just after completion [23]. Although they found that the ventilation system was effective for reducing VOC levels, their data showed much higher concentrations just after completion than we observed.

In addition, our previous study showed that the chemical concentrations in seven houses in the same development considered in this study decreased substantially after one month [17], so we expect that the VOC levels in the houses examined here would also have reduced further after a month. According to the development company that collaborated with us, their newly built houses are only delivered to owners after the following steps are complete: building completion; internal inspection; an owner check; repair work; and final delivery. This normally takes 3–4 weeks, so the IAQ of these houses poses a sufficiently low chemical exposure risk at the time of delivery.

Since building materials constitute an important source of VOC emissions, especially in newly built houses, the houses studied here were built using carefully selected materials. Almost all the materials had their emission rates measured via chamber tests, and low-emission materials were selected and used. In addition, all 49 houses were equipped with 24-h mechanical ventilation systems. We measured the ventilation rates in each of the living rooms and found them to range from 0.5–2.3 air changes per hour. In particular, all the values exceeded the standard specified in the amended Building Standard Law set by the MLIT of Japan (0.5 air changes h). Such systems have been reported as being useful for reducing the VOC concentrations in new houses [24], and we also observed beneficial effects in the seven houses of our previous study [17]. There are two types of ventilation systems, i.e., passive ventilation such as opening a window and active ventilation or mechanical ventilation, but it is desirable to install mechanical ventilation for maintaining a certain number of ventilation rates and preventing crime.

**Figure 1:**
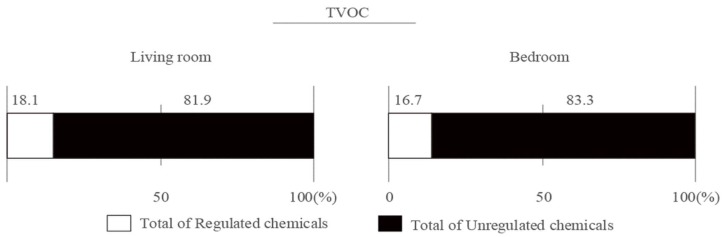
Proportions of regulated and unregulated compounds in the living rooms and bedrooms.

This study has shown that, even just after completion, VOC concentrations can be sufficiently low if we use carefully selected low-emission materials. There are some reports on the health risks in indoor environments, which are often related to the poor IAQ caused by a variety of factors, including chemicals and microbiological contaminants [24,25,26]. This implies that residential houses with good IAQ and those which do not have adverse health effects on humans can be constructed by selecting building materials on the basis of their emission rates. In addition, we also observed that unregulated substances contributed greatly to the TVOC concentrations, reaching approximately 81.9–83.3% immediately after completion (Figure 1). We believe that some of these were substitutes for regulated solvents. In Japan, the VOC concentrations of unregulated compounds tend to be higher than those of regulated ones [27]. Among them some compounds, such as 2-ethyl-1-hexanol, 2-butanone, ethyl acetate and butyl acetate, have been assessed [26] or have TLVs and LCI values [16]. There still exist, however, compounds that have not been assessed nor have guideline values. To reduce the risk of indoor chemical exposure, we need to minimize the concentrations of such unregulated compounds. Thus, we need to identify their sources, and also monitor which materials are being used at worksites and in what quantities.

## 5. Conclusions

By measuring the concentrations of chemical compounds in new houses before furniture and household goods were moved in, we were able to isolate the effect of building materials on IAQ, including the VOC concentrations. The IAQs we observed in newly completed houses in this study show that it may be possible to design houses with low TVOC by carefully considering the materials used. Thus, we believe that it is important to carefully select low-emission building materials to reduce the residents’ exposure to indoor chemicals. In addition, we also believe that active ventilation, which was effective in our previous study, i.e., the installation of a 24-h mechanical ventilation system is equally important. During the next stage of our project, we plan to (a) further explore and analyze IAQ and (b) propose that safe building materials be labeled and listed in a newly created database in Japan.

## Figures and Tables

**Table 1:** Indoor environments.

	Living Room					Bedroom				
	mean	SD (±)	median	max	min	mean	SD (±)	median	max	min
Temp (**°C**)	24.1	4.5	24.0	31.3	7.3	25.5	4.4	25.6	33.2	8.0
Relative humidity (%)	62.7	10.4	64.6	82.2	24.6	58.4	10.3	60.4	77.5	24.4
Ventilation rates (per hour)	1.0	0.4	0.9	2.3	0.5	-	-	-	-	-

-: Not measured.

**Table 2:** Concentrations and frequencies of 13 regulated compounds and the TVOC, including a comparison with the maximum values recommended by the MHLW of Japan.

			Living Room					Bedroom				
	LOQ ^(b)^	Guideline Values ^(c)^	Mean (±SD)	Median	Max	Min	Frequency	Mean (±SD)	Median	Max	Min	Frequency
Compounds ^(a)^	(μg/m^3^)	(μg/m^3^)	μg/m^3^	μg/m^3^	μg/m^3^	μg/m^3^	%	μg/m^3^	μg/m^3^	μg/m^3^	μg/m^3^	%
Toluene	1.0	200	12 (11)	8	64	2	98	10 (8.5)	8	46	2	100
Ethylbenzene	1.0	3800	7 (8.6)	5	60	<1.0 ^(^^d)^	98	7 (7.3)	4	31	<1.0	98
Xylene	1.0	870	5 (5.9)	4	35	<1.0	98	6 (5.7)	4	22	<1.0	98
Styrene	1.0	220	6 (7.2)	3	34	<1.0	98	3 (4.8)	2	27	<1.0	69
*p*-Dichlorobenzene	1.0	240	<1.0	<1.0	5.0	<1.0	27	<1.0	<1.0	4.3	<1.0	29
Tetradecane	1.0	330	3 (1.9)	2	10	<1.0	84	3 (2.7)	3	15	<1.0	84
Formaldehyde	1.0	100	15 (6.5)	13	35	3	100	14 (6.4)	13	32	4	100
Acetaldehyde	1.0	48	24 (10)	20	47	6	100	20 (10)	16	46	5	100
Fenobucarb	0.001	33	<0.001 ^(^^e)^	<0.001	<0.001	<0.001	0.0	<0.001	<0.001	<0.001	<0.001	0.0
Diazinon	0.001	0.29	<0.001	<0.001	<0.001	<0.001	0.0	<0.001	<0.001	<0.001	<0.001	0.0
Dibutyl phthalate	0.07	220	0.1 (0.1)	0.1	0.6	<0.07 ^(^^f)^	96	0.1 (0.2)	0.1	1	<0.07	96
Chlorpyrifos	0.001	1.0	<0.001	<0.001	<0.001	<0.001	0.0	<0.001	<0.001	<0.001	<0.001	0.0
Bis(2-ethylhexyl) phthalate	0.15	120	0.1 (0.1)	0.1 (0.1)	0.4	<0.15 ^(^^g)^	98	0.2 (0.2)	0.1	0.7	<0.15	98
TVOC ^(h)^			389 (112)	390	602	139		390 (123)	396	654	146	

(a) All the compounds were calculated by individual response factor; (b) LOQ: limit of quantitation; (c) The MHLW of Japan set non-binding guideline values for 13 chemicals (six VOCs, two aldehydes and five SVOCs); (d), (e), (f), (g) < LOQ; (h) TVOC: Total concentration of 55 VOCs, 14 carbonyl compounds, and 22 SVOCs

**Table 3:** Comparison of concentrations of 13 VOCs and TVOC in indoor which have been set by MHLW.

Compounds ^(a)^	Our study (2015)		Park et al. (2006)	Jarnstrom et al. (2006)	Shin et al. (2012)	Noguchi et al. (2016)	Onuki et al. (2009)
	Mean (±SD)		Mean (±SD)	Mean (±SD)	Mean (±SD)	Mean (±SD)	Median
	Living room	Bedroom					
Toluene	12 (11)	10 (8.5)	27 (56)	20	184 (112)	16.9	29.8
Ethylbenzene	7 (8.6)	7 (7.3)	20 (68)	29 (42)	8.2 (3.8)	6.4	12.5
Xylene	5 (5.9)	6 (5.7)	30 (63)	110	16.8	14.3	18.0
Styrene	6 (7.2)	3 (4.8)	64 (190)	3 (4)	2.7 (2.5)	60	5.9
*p*-Dichlorobenzene	<1.0	<1.0	87 (126)	nm	6.7 (4.1)	nm	0.6
Tetradecane	3 (1.9)	3 (2.7)	nm ^(c)^	5 (8)	0.5 (0.5)	nm	0.9
Formaldehyde	15 (6.5)	14 (6.4)	134 (93)	19 (26)	0.05 (0.03) (ppm)	9.5	15.9
Acetaldehyde	24 (10)	20 (10)	nm	nm	nm	33	27.7
Fenobucarb	<0.001	<0.001	nm	nm	nm	nm	nm
Diazinon	<0.001	<0.001	nm	nm	nm	nm	nm
Dibutyl phthalate	0.1 (0.1)	0.1 (0.2)	nm	nm	nm	nm	nm
Chlorpyrifos	<0.001	<0.001	nm	nm	nm	nm	nm
Bis(2-ethylhexyl) phthalate	0.1 (0.1)	0.2 (0.2)	nm	nm	nm	nm	nm
TVOC ^(d),(e),(f)^	389 (112) ^(d)^	390 (123) ^(d)^	328 (720) ^(e)^	780 (1103) ^(f)^	nm	2327 ^(g)^	1280 ^(e)^
Regulated chemicals	70.0	65.0	nm	nm	nm	nm	137
Unregulated chemicals	319	325	nm	nm	nm	nm	1540

(a) All the compounds were calculated by individual response factor. (b) LOQ: limit of quantitation. (c) nm: not measured; (d) TVOC: Total concentrations of 55VOCs, 14 carbonyl compounds and 22 SVOCs. (e) TVOC: Sum of VOCs (26). (f) TVOC: Total concentrations calculated as toluene between hexane and hexadecane. (g) TVOC: Total concentrations calculated as toluene between hexane and hexadecane and carbonyl compounds.

## Supplementary Materials

The following are available online at https://www.mdpi.com/1660-4601/16/21/4142/s1, Table S1: VOC concentrations in the living rooms and bedrooms, Table S2: Carbonyl compounds in the living rooms and bedrooms, Table S3: SVOC concentrations in the living rooms and bedrooms.
