# Supplementary material for: Indoor Air Quality Analysis of Newly Built Houses
Source: Int J Environ Res Public Health. 2019 Oct 28;16(21):4142. doi: 10.3390/ijerph16214142 (PMC6862697; doi:10.3390/ijerph16214142)
Supplement: Supplementary file 1 [file ijerph-16-04142-s001.pdf]

Table S1: The concentrations of VOCs in living room and bed room

	living room					bed room				
VOCs [a)]	mean (±SD) µg/m³	median (µg/m³)	max (µg/m³)	min (µg/m³)	frequency %	mean (±SD) µg/m³	median (µg/m³)	max (µg/m³)	min (µg/m³)	frequency (%)
2-Propanol	< 1.0[b)]	< 1.0	5.1	< 1.0	31	< 1.0	< 1.0	8.9	< 1.0	29
Pentane	3.6(6.1)	1.5	35	< 1.0	63	< 1.0	< 1.0	6.3	< 1.0	22
Mthyl acetate	7.8(4.3)	6.8	18	1.2	98	5.9(4.7)	3.8	19	< 1.0	98
Dichloromethane	< 1.0	< 1.0	6.0	< 1.0	22	< 1.0	< 1.0	3.8	< 1.0	14
1-Propanol	< 1.0	< 1.0	8.6	< 1.0	12	< 1.0	< 1.0	4.4	< 1.0	10
Ethyl acetate	13.8(6.7)	13	31	2.5	100	16.5(20.1)	13	260	2.5	100
Hexane	< 1.0	< 1.0	7.5	< 1.0	41	1.1(1.9)	< 1.0	9.1	< 1.0	33
Chloroform	< 1.0	< 1.0	< 1.0	< 1.0	0.0	< 1.0	< 1.0	< 1.0	< 1.0	0.0
1,2-Dichloroethane	< 1.0	< 1.0	< 1.0	< 1.0	0.0	< 1.0	< 1.0	< 1.0	< 1.0	0.0
2,4-Dimethylpentane	< 1.0	< 1.0	< 1.0	< 1.0	0.0	< 1.0	< 1.0	< 1.0	< 1.0	0.0
1,1,1-Trichloroethane	< 1.0	< 1.0	< 1.0	< 1.0	0.0	< 1.0	< 1.0	< 1.0	< 1.0	0.0
Butanol	12.6(7.8)	11	34	2.5	100	16.2(9.1)	17	57	3.0	100
Benzene	< 1.0	< 1.0	3.9	< 1.0	47	< 1.0	< 1.0	4.4	< 1.0	27
Carbon tetrachloride	< 1.0	< 1.0	< 1.0	< 1.0	0.0	< 1.0	< 1.0	< 1.0	< 1.0	0.0
Cyclohexane	< 1.0	< 1.0	3.0	< 1.0	18	< 1.0	< 1.0	3.7	< 1.0	2.0
1,2-Dichloropropane	< 1.0	< 1.0	< 1.0	< 1.0	0.0	< 1.0	< 1.0	< 1.0	< 1.0	0.0
Bromodichloromethane	< 1.0	< 1.0	< 1.0	< 1.0	0.0	< 1.0	< 1.0	1.1	< 1.0	2.0
Trichloroethylene	< 1.0	< 1.0	2.6	< 1.0	12	< 1.0	< 1.0	2.6	< 1.0	7.8
Isooctane	< 1.0	< 1.0	< 1.0	< 1.0	0.0	< 1.0	< 1.0	< 1.0	< 1.0	0.0
Heptane	< 1.0	< 1.0	9.4	< 1.0	31	< 1.0	< 1.0	2.6	< 1.0	14
4-Methyl-2-pentanone(MIBK)	14.8(9.9)	12	50	1.6	100	20.1(12.4)	22	71	1.8	100
Methylcyclohexane	2.7(11.6)	1.0	30	< 1.0	51	3.7(13)	< 1.0	110	< 1.0	18
Dibromochloromethane	< 1.0	< 1.0	< 1.0	< 1.0	0.0	< 1.0	< 1.0	1.0	< 1.0	2.0
Butyl acetate	10.7(6.3)	10	34	1.7	100	16.7(19)	12	170	1.7	100
Octane	< 1.0	< 1.0	11.0	< 1.0	7.8	< 1.0	< 1.0	1.2	< 1.0	3.9
Tetrachloroethylene	< 1.0	< 1.0	< 1.0	< 1.0	2.0	< 1.0	< 1.0	< 1.0	< 1.0	0.0

2-Butoxyethanol	3.5(3.9)	2.4	22	< 1.0	92	3.2(3.4)	2.6	21	< 1.0	82
Nonane	1.0(2.4)	< 1.0	16	< 1.0	49	< 1.0	< 1.0	7.7	< 1.0	33
Tricyclene	< 1.0	< 1.0	< 1.0	< 1.0	0.0	< 1.0	< 1.0	< 1.0	< 1.0	0.0
α-Pinene	13.2(7.4)	12	35	3.1	100	14.8(10.6)	16	66	1.9	100
3-Ethyltoluene	< 1.0	< 1.0	21	< 1.0	31	< 1.0	< 1.0	4.3	< 1.0	20
Camphene	< 1.0	< 1.0	1.6	< 1.0	14	< 1.0	< 1.0	1.4	< 1.0	2.0
4-Ethyltoluene	< 1.0	< 1.0	14	< 1.0	10	< 1.0	< 1.0	2.1	< 1.0	10
1,3,5-Trimethylbenzen	< 1.0	< 1.0	13	< 1.0	14	< 1.0	< 1.0	3.2	< 1.0	14
2-Ethyltoluene	< 1.0	< 1.0	12	< 1.0	12	< 1.0	< 1.0	2.5	< 1.0	12
β-Pinene	3.6(2.7)	3.5	10	< 1.0	90	2.5(1.9)	2.5	7.6	< 1.0	80
1,2,4-Trimethylbenzen	1.9(3.6)	1.2	21	< 1.0	78	1.9(2.7)	1.2	11	< 1.0	61
D4	1.7(1.9)	1.5	8.0	< 1.0	75	< 1.0	< 1.0	7.1	< 1.0	43
Decane	5.2(4.8)	3.6	25	< 1.0	94	4.5(5.7)	2.2	87	< 1.0	82
Isododecane	2.3(2.3)	1.7	9.3	< 1.0	80	2.4(5.5)	1.2	53	< 1.0	51
2-Ethyl-1-hexanol	15.6(8.1)	13	37	1.8	100	14.3(7.3)	14	71	1.7	100
3-Carene	7.4(7.3)	5.1	38	< 1.0	98	5.6(5.5)	4.1	22	< 1.0	92
1,2,3-Trimethylbenzen	< 1.0	< 1.0	12	< 1.0	18	< 1.0	< 1.0	3.0	< 1.0	14
p-Cymene	1.2(1.3)	1.2	5.2	< 1.0	75	< 1.0	< 1.0	3.0	< 1.0	39
Limonene	3.7(3.3)	3.0	14	< 1.0	92	2.2(2.0)	2.4	13	< 1.0	69
4-Ethyl-1,2-dimethylbenzen	< 1.0	< 1.0	2.9	< 1.0	18	< 1.0	< 1.0	1.7	< 1.0	14
Undecane	88.5(29.4)	91	160	22	100	74.3(35.3)	80	150	3.5	100
1,2,4,5-Tetramethylbenzen	< 1.0	< 1.0	1.2	< 1.0	3.9	< 1.0	< 1.0	1.4	< 1.0	2.0
D5	3.5(2.2)	3.6	8.8	< 1.0	98	2.5(2.2)	2.3	14	< 1.0	76
Dodecane	1.8(1.1)	1.7	4.8	< 1.0	88	3.7(14)	1.8	98	< 1.0	80
Tridecane	< 1.0	< 1.0	13	< 1.0	45	2.1(8.9)	< 1.0	63	< 1.0	41
D6	4.1(2.2)	3.3	9.1	< 1.0	98	3.9(3.0)	3.2	15	< 1.0	96
Texanol	11.4(9.2)	10	50	2.0	100	18(25)	9.2	120	1.5	100
Pentadecane	< 1.0	< 1.0	2.2	< 1.0	12	< 1.0	< 1.0	3.4	< 1.0	16
Hexadecane	< 1.0	< 1.0	2.1	< 1.0	25	< 1.0	< 1.0	2.4	< 1.0	22

a) All the compounds were calculated by individual response factor.

b) < LOQ

Table S2: The concentrations of Carbonyl compounds in living room and bed room

	mean (±SD)	median	max	Min	frequency	mean (±SD)	median	max	min	frequency
	**living room**					**bed room**				
Carbonyl compounds [a)]	μg/m³	(μg/m³)	(μg/m³)	(μg/m³)	%	μg/m³	(μg/m³)	(μg/m³)	(μg/m³)	(%)
Acetone	22(9.1)	22	44	3.1	100	37.7(21.6)	35	100	2.1	100
2-Furanacrolein	< 1.0 [b)]	< 1.0	< 1.0	< 1.0	0	< 1.0	< 1.0	< 1.0	< 1.0	0
Propionaldehyde	2.2(1.3)	1.9	4.8	< 1.0	90	1.8(1.1)	1.7	4.2	< 1.0	84
2-Butanone	17(8.2)	14	42	5.0	100	14.7(11.4)	12	55	2.6	100
Butanal	1.4(1.3)	1.3	4.2	< 1.0	80	1.1(1.0)	1.1	2.9	< 1.0	63
Cyclohexanone	9.3(7.3)	7.5	29	< 1.0	96	8.9(8.5)	6.2	53	< 1.0	98
Benzaldehyde	2.8(2.4)	2.6	9.3	< 1.0	80	3.4(3.2)	2.9	13	< 1.0	76
Pentanal	2.7(2.1)	2.2	8.4	< 1.0	92	1.7(1.7)	1.4	6.3	< 1.0	69
Tolualdehyde	< 1.0	< 1.0	< 1.0	< 1.0	0.0	< 1.0	< 1.0	7.6	< 1.0	2.0
Hexaldehyde	12(7.6)	10	30	< 1.0	98	9.1(5.8)	8.0	24	< 1.0	98
Heptanal	< 1.0	< 1.0	2.1	< 1.0	61	< 1.0	< 1.0	2.5	< 1.0	53
Octanol	< 1.0	1.0	2.8	< 1.0	67	1(1.0)	1.2	3.9	< 1.0	63
Nonanal	6.1(3.7)	5.7	15	< 1.0	96	7.6(4.5)	6.3	22	1.3	100
Decanal	1.3(1.5)	1.3	7.4	< 1.0	78	1.7(1.1)	1.6	7.2	< 1.0	92

a) All the compounds were calculated by individual response factor.
b) < LOQ

Table S3: The concentrations of SVOCs in living room and bed room

SVOCs [a]	mean (±SD) µg/m³	median (µg/m³)	max (µg/m³)	min (µg/m³)	frequency %	mean (±SD) µg/m³	median (µg/m³)	max (µg/m³)	min (µg/m³)	frequency (%)
Trimethyl phosphate	< 0.001 [b]	< 0.001	< 0.001	< 0.001	0.0	< 0.001	< 0.001	< 0.001	< 0.001	0.0
Triethyl phosphate	< 0.001	< 0.001	< 0.001	< 0.001	0.0	< 0.001	< 0.001	< 0.001	< 0.001	0.0
DDVP	< 0.001	< 0.001	< 0.001	< 0.001	0.0	< 0.001	< 0.001	< 0.001	< 0.001	0.0
Tripropyl Phosphate	< 0.001	< 0.001	< 0.001	< 0.001	0.0	< 0.001	< 0.001	< 0.001	< 0.001	0.0
Diethyl phthalate	0.02(0.02)	0.02	0.12	< 0.001	63	0.03(0.03)	0.02	0.15	< 0.001	67
Tributyl phosphate	< 0.001	< 0.001	< 0.001	< 0.001	0.0	< 0.001	< 0.001	< 0.001	< 0.001	0.0
TCEP	< 0.001	< 0.001	< 0.001	< 0.001	0.0	< 0.001	< 0.001	< 0.001	< 0.001	0.0
DIPROPYL PHTHALATE	< 0.001	< 0.001	< 0.001	< 0.001	0.0	< 0.001	< 0.001	< 0.001	< 0.001	0.0
TCIPP	< 0.001	< 0.001	0.2	< 0.001	2.0	< 0.001	< 0.001	0.2	< 0.001	2.0
DICHLOFENTHION	< 0.001	< 0.001	< 0.001	< 0.001	0.0	< 0.001	< 0.001	< 0.001	< 0.001	0.0
Chlorpyrifos-methyl	< 0.001	< 0.001	< 0.001	< 0.001	0.0	< 0.001	< 0.001	< 0.001	< 0.001	0.0
Fenitrothion	< 0.001	< 0.001	< 0.001	< 0.001	0.0	< 0.001	< 0.001	< 0.001	< 0.001	0.0
Malathion	< 0.001	< 0.001	< 0.001	< 0.001	0.0	< 0.001	< 0.001	< 0.001	< 0.001	0.0
DI-N-PENTYL PHTHALATE-D4	< 0.001	< 0.001	< 0.001	< 0.001	0.0	< 0.001	< 0.001	< 0.001	< 0.001	0.0
TDCPP	< 0.001	< 0.001	< 0.001	< 0.001	0.0	< 0.001	< 0.001	< 0.001	< 0.001	0.0
DI-N-HEXYL PHTHALATE	< 0.001	< 0.001	< 0.001	< 0.001	0.0	< 0.001	< 0.001	< 0.001	< 0.001	0.0
Butyl benzyl phthalate	< 0.001	< 0.001	< 0.001	< 0.001	0.0	< 0.001	< 0.001	< 0.001	< 0.001	0.0
TBEP	< 0.001	< 0.001	< 0.001	< 0.001	0.0	< 0.001	< 0.001	< 0.001	< 0.001	0.0
TPhP	< 0.001	< 0.001	< 0.001	< 0.001	0.0	< 0.001	< 0.001	< 0.001	< 0.001	0.0
Pyridaphenthion	< 0.001	< 0.001	< 0.001	< 0.001	0.0	< 0.001	< 0.001	< 0.001	< 0.001	0.0
Dicyclohexyl phthalate	< 0.001	< 0.001	< 0.001	< 0.001	0.0	< 0.001	< 0.001	< 0.001	< 0.001	0.0
Tritolyl phosphate	< 0.001	< 0.001	< 0.001	< 0.001	0.0	< 0.001	< 0.001	< 0.001	< 0.001	0.0

a) All the compounds were calculated by individual response factor.

b) < LOQ
